# Watching the Effects of Gravity. Vestibular Cortex and the Neural Representation of “Visual” Gravity

**DOI:** 10.3389/fnint.2021.793634

**Published:** 2021-12-01

**Authors:** Sergio Delle Monache, Iole Indovina, Myrka Zago, Elena Daprati, Francesco Lacquaniti, Gianfranco Bosco

**Affiliations:** ^1^UniCamillus—Saint Camillus International University of Health Sciences, Rome, Italy; ^2^Laboratory of Neuromotor Physiology, IRCCS Santa Lucia Foundation, Rome, Italy; ^3^Department of Biomedical and Dental Sciences and Morphofunctional Imaging, University of Messina, Messina, Italy; ^4^Center for Space Biomedicine, University of Rome “Tor Vergata”, Rome, Italy; ^5^Department of Civil and Computer Engineering, University of Rome “Tor Vergata”, Rome, Italy; ^6^Department of Systems Medicine, University of Rome “Tor Vergata”, Rome, Italy

**Keywords:** internal model, vestibular network, neuroimaging, TMS, connectomics, psychophysics, insula, temporo-parietal junction (TPJ)

## Abstract

Gravity is a physical constraint all terrestrial species have adapted to through evolution. Indeed, gravity effects are taken into account in many forms of interaction with the environment, from the seemingly simple task of maintaining balance to the complex motor skills performed by athletes and dancers. Graviceptors, primarily located in the vestibular otolith organs, feed the Central Nervous System with information related to the gravity acceleration vector. This information is integrated with signals from semicircular canals, vision, and proprioception in an ensemble of interconnected brain areas, including the vestibular nuclei, cerebellum, thalamus, insula, retroinsula, parietal operculum, and temporo-parietal junction, in the so-called vestibular network. Classical views consider this stage of multisensory integration as instrumental to sort out conflicting and/or ambiguous information from the incoming sensory signals. However, there is compelling evidence that it also contributes to an internal representation of gravity effects based on prior experience with the environment. This *a priori* knowledge could be engaged by various types of information, including sensory signals like the visual ones, which lack a direct correspondence with physical gravity. Indeed, the retinal accelerations elicited by gravitational motion in a visual scene are not invariant, but scale with viewing distance. Moreover, the “visual” gravity vector may not be aligned with physical gravity, as when we watch a scene on a tilted monitor or in weightlessness. This review will discuss experimental evidence from behavioral, neuroimaging (connectomics, fMRI, TMS), and patients’ studies, supporting the idea that the internal model estimating the effects of gravity on visual objects is constructed by transforming the vestibular estimates of physical gravity, which are computed in the brainstem and cerebellum, into internalized estimates of virtual gravity, stored in the vestibular cortex. The integration of the internal model of gravity with visual and non-visual signals would take place at multiple levels in the cortex and might involve recurrent connections between early visual areas engaged in the analysis of spatio-temporal features of the visual stimuli and higher visual areas in temporo-parietal-insular regions.

## Introduction

Gravity represents a physical invariant of the Earth environment to which all species, including ours, have adapted through evolution. A clear exemplification of such adaptation is represented by the lack of conscious effort with which gravity effects are taken into account when controlling most motor behaviors, ranging from the seemingly simple task of maintaining balance during gait to the complex motor skills performed by professional athletes, acrobats, and ballet dancers. Moreover, gravity cues provide an absolute spatial reference, crucial for navigation and, more generally, for spatial perception (Jeffery et al., 2013; Angelaki et al., 2020). Information about gravity is relayed to the Central Nervous System (CNS) by multiple sensory sources, namely, the vestibular organs, the retina, skin, muscle, tendon, and visceral receptors (Mittelstaedt, 1992). In particular, vestibular otolith organs (saccule and utricle) are considered the main graviceptors. Hair cells in the neuroepithelium of their maculae are stimulated by gravito-inertial accelerations, thereby signaling head accelerations due to linear inertial motion as well as to changes of head orientation relative to gravity (Fernández and Goldberg, 1976). Remarkably, this ambiguity about the nature of the accelerative force inherent to the otoliths’ afferent signals is tackled early in the processing of vestibular information. In fact, during dynamic head tilts, gravito-inertial accelerations signaled by the otoliths can be disambiguated by filtering the otolith signals (Mayne, 1974) and/or combining them with signals from the semicircular canals in the vestibular nuclei and the cerebellum (Glasauer, 1992; Angelaki et al., 1999; Merfeld et al., 1999; Mackrous et al., 2019). Thus, Purkinje cells in the caudal vermis integrate otolith and semicircular canal inputs during passively applied self-motion (see Angelaki and Cullen, 2008). A subset of these neurons represents head orientation relative to gravity, whereas another subset preferentially encodes translational self-motion (Laurens et al., 2013). The gravity-driven responses are canceled for self-generated movements, indicating that the brain builds a dynamic prediction of the sensory consequences of gravity to ensure postural and perceptual stability (Mackrous et al., 2019). Information from the vestibular nuclei and cerebellum is relayed and processed in several regions of the brain and spinal cord, giving rise to sensations and movements (Angelaki and Cullen, 2008).

Although vestibular signals may be combined with visual and somatosensory information as early as in the vestibular nuclei (Waespe and Henn, 1978; Carleton and Carpenter, 1983; Büttner-Ennever, 1992; Barmack, 2003; Shinder and Taube, 2010; Cullen, 2012), vestibular only neurons, projecting from the vestibular nuclei to the thalamus/cortex, do not receive under normal conditions visual and/or somatosensory inputs. These latter inputs, however, can be un-masked along with efference copy signals after labyrinthectomy (Cullen et al., 2009; Sadeghi et al., 2009, 2011, 2012). Instead, more extensive multisensory integration takes place at a higher processing level, within several interconnected subcortical structures (such as the thalamus) and cortical areas around the sylvian sulcus, namely, the insula, the retroinsula, the parietal operculum, the temporo-parietal junction, forming the so-called vestibular network (Guldin and Grüsser, 1998; Lopez and Blanke, 2011). One particular functional aspect of the multisensory integration process occurring in the vestibular network, which the present review article will focus on, is inherent to the notion that visual, as well as somatosensory signals, can embed information about gravity. In this respect, the contribution of the somatosensory system to graviception can be inferred by considering the upright stance condition where the effects of gravity on the body determine the distribution of pressure forces on the feet soles, sensed by cutaneous receptors, and that of limb extensor muscles loads, sensed by Golgi Tendon Organs. On the contrary, for the visual system, there are several factors that make extracting gravity-related information from retinal signals a less straight-forward process. First, despite the fact that gravitational acceleration is quasi-constant on Earth (its magnitude varies by <1% and its vertical deflection by <0.05° at different latitudes or altitudes), retinal accelerations elicited by visual targets moving along the fronto-parallel plane under gravity are hardly constant since they scale inversely with viewing distance. Secondly, for motion-in-depth, such as when an object accelerated by gravity approaches the viewer (i.e., projectile motion), the retinal speed (rate of change of image size, elevation, and disparity) is related non-linearly to the object speed in world coordinates. Thirdly, besides differences in magnitude between physical and retinal accelerations, the direction of “visual” gravity is not invariably aligned with that of physical gravity, as in the case of watching a scene on a tilted monitor or in weightlessness. Finally, as a further complication, the visual system is poorly sensitive to arbitrary accelerations, especially over short time windows (Bennett et al., 2007). Thus, for both fronto-parallel motion (Werkhoven et al., 1992; Brouwer et al., 2002) and motion in depth (Trewhella et al., 2003; Lee et al., 2019), the Weber fractions of acceleration discrimination (i.e., the ratio of just noticeable difference to the absolute value of acceleration, and thus a measure of perceptual precision) are more than five times worse than those of speed discrimination.

Unsurprisingly, the motor system generally does not account well for arbitrary visual accelerations, as shown by manual interceptive responses to targets moving along a horizontal line with different positive or negative accelerations (Port et al., 1997; Benguigui et al., 2003) or by ocular tracking responses to accelerated targets (Bennett and Barnes, 2006). In these situations, spatial and temporal errors tend to be relatively small for low accelerations but increase steeply with increasing accelerations. It is worth noting that the motion accelerations imposed to the visual targets in these experiments were considerably lower than the gravity acceleration and, by extrapolating these results, one might expect timing errors of about 400 ms for targets accelerated by gravity!

However, people exhibit remarkable accuracy and precision when interacting with targets accelerated by gravity. In fact, small timing and spatial errors are generally observed when subjects catch or punch a ball in free-fall from different heights (Lacquaniti and Maioli, 1989; Zago et al., 2004, 2011a,b; Indovina et al., 2005; Katsumata and Russell, 2012; Brenner and Smeets, 2015) or approaching in projectile motion (Russo et al., 2017). Interestingly, the greater accuracy at intercepting targets accelerated by gravity is also evident when a substantial portion of the target path is occluded from vision, implying that visual extrapolation mechanisms take into account natural gravity effects on objects’ motion (Bosco et al., 2012; La Scaleia et al., 2015). Alike manual interception studies, ocular tracking experiments have shown significantly greater accuracy following target motion modeled according to natural kinematics (gravity and air drag) compared to arbitrary kinematics (hypo- or hypergravity; Diaz et al., 2013a, b; Delle Monache et al., 2015, 2019; Jörges and López-Moliner, 2019; Meso et al., 2020). Visual effects of gravity are taken into account, although with variable precision (see below), also in perceptual tasks that do not necessarily involve the production of motor response timed to the target motion, such as the discrimination of motion duration for targets shifting along the vertical (Moscatelli and Lacquaniti, 2011; Torok et al., 2019; Gallagher et al., 2020), time-to-passage estimation during virtual self-motion (Indovina et al., 2013a), visuomotor synchronization (Zhou et al., 2020), naturalness judgments of motion under gravity (La Scaleia et al., 2014, 2020; Ceccarelli et al., 2018), speed discrimination of targets moving in different directions (Moscatelli et al., 2019), and interpretation of biological motion (Chang and Troje, 2009; Maffei et al., 2015).

How does the visual system account for gravity acceleration, given that image accelerations are poorly discriminated (see above)? According to a current hypothesis, an internal model mimics the expected gravity effects on visual targets (Lacquaniti and Maioli, 1989; Tresilian, 1997; Zago et al., 2008, 2009; Lacquaniti et al., 2013, 2014, 2015; Jörges and López-Moliner, 2017). Compatible with this idea, erroneous expectations of Earth’s gravity effects are evident in the timing of interceptive responses to visual targets moving vertically downward at a constant speed, due to either real weightlessness in a spacelab (McIntyre et al., 2001) or simulated weightlessness in the laboratory (Zago et al., 2004; Bosco et al., 2012; Russo et al., 2017; La Scaleia et al., 2020). These findings suggest that the brain is able to build an* a priori* knowledge of gravity effects based on innate mechanisms and/or learning with daily experience. Thus, in order to produce accurate response timing when intercepting targets accelerated by gravity, this internal model of gravity effects is combined with online visual signals about the target position and velocity (Zago et al., 2008, 2009; Lacquaniti et al., 2013, 2014, 2015). Moreover, in order to map visual information between retinal and world coordinates, the visual effects of gravity on a moving target can be interpreted by combining information about the rate of change of retinal image with binocular (stereo, vergence) and monocular (familiar size, vertical and horizontal scene contours, perspective, shading, texture gradient, lighting, etc) cues (Zago et al., 2009).

**Figure 1:**
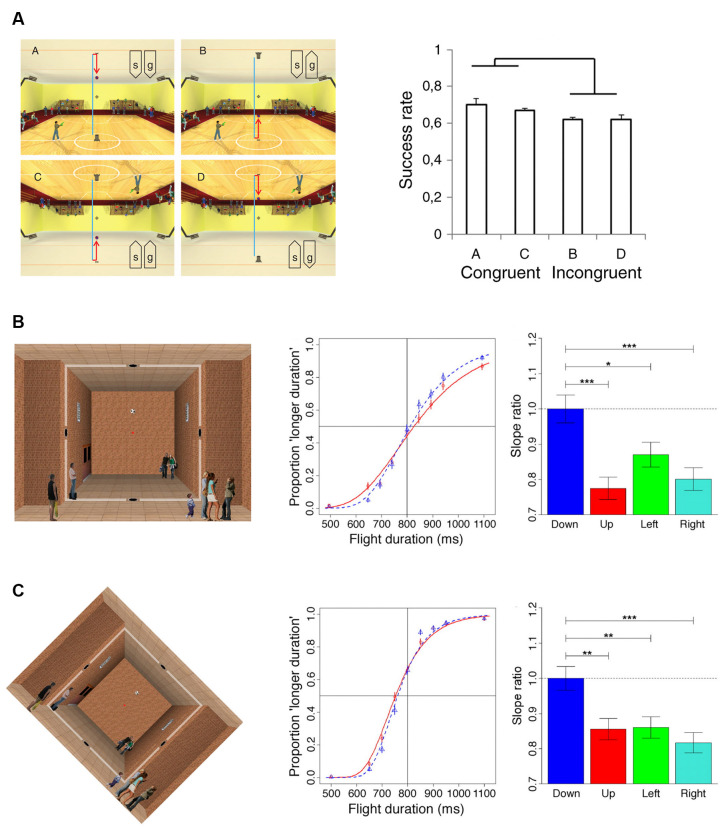
Results of psychophysical experiments manipulating the direction of the “visual” gravity vector relative to physical gravity. **(A)**The four panels on the left side illustrate the scenarios (indicated with “a”, “b”, “c”, “d”) employed in Experiment 1 of Zago et al. (2011a). A ball was launched vertically and bounced back hitting the opposite side. The ball decelerated until bouncing (blue trajectory), and then it accelerated (red trajectory). Participants pressed a button to trigger the standing character to shoot a bullet to hit the ball at the interception point (indicated by the crosshair). The orientation of the scene (“s”) and the direction of the simulated gravity acting on the target (“g”) were manipulated in different blocks of trials: (upper left, scenario “a”) normal scene orientation and gravity, (upper right, scenario “b”) normal scene orientation but inverted target gravity, (lower left, scenario “c”) both scene and gravity were inverted, and (lower right, scenario “d”) inverted scene and normal target gravity. The panel on the right side illustrates the interception scores (success rates) observed for the four scenario conditions described above. **(B)** The leftmost panel illustrates the background scene and the visual target of Experiment 1 of Moscatelli and Lacquaniti (2011). The soccer ball moved at constant acceleration between two holes located on opposite sides of the room and, in different blocks of trials, along four possible directions: downward, upward, rightward, or leftward. Ball kinematics was congruent with the effects of gravity only in the downward direction. Participants maintained fixation throughout the trial on the red dot at the center of the scene. The middle panel illustrates the psychometric functions for downward (blue) and upward motion (red), obtained by pooling data of the seven participants of Experiment 1. The rightmost panel shows the precision of discrimination for the four directions of motion quantified by the slope of a Generalized linear mixed model fitted to the subject population data. Slopes were normalized to the values obtained for the downward direction. Error bars refer to ± 1 SD; ***, ** and * denote significant differences at *p* < 0.001, *p* < 0.01 and *p* < 0.05 level, respectively. **(C)** The leftmost panel illustrates the visual scene used for Experiment 6 of Moscatelli and Lacquaniti (2011), which was identical to Experiment 1, except for the 45 degrees clockwise rotation of the computer display. The middle and rightmost panels illustrate the results of this experiment with the same format as the corresponding panels in **(B)**.

We mentioned earlier that the direction of visual gravity is not always coincident with that of physical gravity. Some insight on how this discrepancy is dealt with has come from the work of Zago et al. (2011a). This study manipulated the alignment between the visual gravity vector and stationary visual cues, as well as relative to the orientation of the observer and of the physical gravity vector. Participants pressed a button, which triggered a hitter to intercept targets moving with constant acceleration, scaled to the visual scene so as to be congruent with Earth gravitational acceleration. A factorial design assessed the effects of scene orientation (normal or inverted) and target gravity (normal or inverted). Interception scores were significantly higher when scene direction was concordant with target gravity direction, irrespective of whether both were upright or inverted (Figure 1A). Therefore, the combined influence of visible gravity and structural visual cues can outweigh both physical gravity and viewer-centered cues, yielding to rely instead on the congruence of the apparent physical forces acting on people and objects in the represented visual scene. In another study (Moscatelli and Lacquaniti, 2011), observers judged the duration of motion of a target accelerating over a fixed length path in one of the different directions. The visual motion was presented to participants either over a pictorial scene or a uniform background and while either standing upright or tilted by 45° relative to the computer display and Earth’s gravity. In another experimental condition, observers were upright and the scene was tilted by 45°. Results of these experiments indicated again that the effects of virtual gravity can be represented with respect to a pseudo-vertical direction concordant with the visual scene orientation and discordant with the direction of Earth’s gravity (Figures 1B,C). By applying the model of the vision group at York University (Jenkin et al., 2004; Dyde et al., 2006) to their data, Moscatelli and Lacquaniti (2011) found that a weighted sum of the observer orientation, target motion orientation, and pictorial scene orientation relative to physical gravity could account for the estimated downward of visual gravity, with weighing coefficients of 43, 37, and 20%, respectively. These weightings, however, vary considerably as a function of the specific task and context.

Current evidence indicates that the internal model of gravity effects is qualitative and does not comply with physics exactly. Indeed, as mentioned above, people systematically underestimate the motion duration of constant speed targets descending along the vertical and activate their arm muscles too early to intercept them (McIntyre et al., 2001; Zago et al., 2004). The precision of perceptual judgments of the duration of parabolic motions is independent of whether the target moves according to natural gravity or it shifts at a constant speed (Jörges et al., 2021). A general heuristic that assumes that descending targets or moving as projectiles are affected by gravity might provide information that is generally good enough while requiring much less cognitive processing or visual resources than exact models of physics (Zago et al., 2008; Vicovaro et al., 2021). However, as remarked above, motor actions on targets accelerated by gravity can be strikingly accurate, presumably because of the integration with online sensory information about target motion.

### Behavioral Evidence About Vestibular and Somatosensory Contributions to Modeling The Effects of Gravity on Visual Target Motion

In line with the principle, visual processing of gravitational motion could be independent of the vestibular and somatosensory processing of physical gravity. There is, however, behavioral evidence that this is not the case, since vestibular and somatosensory cues about the head and body orientation help construct a gravity reference for intercepting visual targets. A number of studies have shown that the participant’s posture relative to gravity direction contributes to providing a sense of Up and Down in the interception of targets moving along the vertical (Senot et al., 2005; Le Séac’h et al., 2010; Baurès and Hecht, 2011). In these studies, subjects intercepted a ball approaching from above or below in a virtual scene presented with a head-mounted stereoscopic display. Above (below) was obtained in sitting subjects (Senot et al., 2005) who pitched the head backward (forward) so as to look up (down) toward a virtual ceiling (floor), or in lying subjects (Le Séac’h et al., 2010; Baurès and Hecht, 2011) who looked up (down) while supine (prone). Interception responses were significantly earlier for downward than upward moving targets, consistent with an expectation that downward motion is faster than upward motion under gravity (Senot et al., 2005; Le Séac’h et al., 2010; Baurès and Hecht, 2011). This expectation is naïve because it violates Newtonian mechanics, according to which downward and upward displacements under gravity along a given vertical path have the same duration (in fact, with air resistance, upward is actually faster than downward motion, Timmerman and van der Weele, 1999). Interestingly, also targets shifting downward at constant speed are perceived as moving faster than the same targets moving at the same speed upward or rightward (Moscatelli et al., 2019).

A direct role for vestibular inputs has been shown with parabolic flight experiments where upward and downward motion were tested in weightlessness and on the ground. These experiments showed that the response bias (i.e., earlier responses for downward compared to upward motion) reversed sign between the weightlessness and the ground condition, mirroring the sign reversal of otolith signals at the transition from the hypergravity to the hypogravity phase of the parabolic flight (Senot et al., 2012). Moreover, sound-evoked stimulation of the otolith receptors interferes with the anticipation of gravity effects during visually simulated self-motion in the downward direction (Indovina et al., 2015), and unloading of the otoliths in the weightless conditions of space flight affects Up/Down asymmetries in the perception of self-motion (De Saedeleer et al., 2013).

A quantitative assessment of the role of vestibular and somatosensory cues about the head and body orientation on interception timing was reported by La Scaleia et al. (2019). In their experiment, participants hit a ball rolling in a gutter towards the eyes, resulting in image expansion. The scene was presented in a head-mounted display, without any visual information about gravity direction. In separate blocks of trials, participants were pitched backwards by 20° or 60°, while ball acceleration was randomized across trials to be compatible with rolling down a slope of 20° or 60°. Initially, the timing errors were large, independent of the coherence between ball acceleration and pitch angle, consistent with responses based exclusively on visual information (since visual stimuli were identical at both tilts). At the end of the experiment, however, the timing errors were systematically smaller in the coherent conditions than the incoherent ones. Therefore, practice with the task led to the incorporation of information about head and body orientation relative to gravity for response timing. Such information could have been extracted by combining signals from at least two sources: (1) the background activity and dynamic sensitivity of otolith regular afferents, which are related to the component of the gravitational shear force acting in the plane of the maculae, changed by the static head tilt; and (2) signals from somatosensory (cutaneous, muscle, and tendon) and visceral receptors (in the kidneys, vena cava), which monitor contact forces between the body and the environment, thereby contributing a sense of body orientation.

Visual gravity and information about the actual body posture interact to provide a gravity reference. Purely visual cues from the inclination of the support surface in virtual reality induce locomotor adaptations to counter expected gravity-based changes similar to what happens with real inclinations (Cano Porras et al., 2019). When the task requires aligning a visual line to the vertical in the dark, the so-called subjective visual vertical or SVV (Lacquaniti et al., 2015; Kheradmand and Winnick, 2017), the direction of gravity is estimated by combining retinal cues about the line orientation with vestibular and somatosensory cues about the head and body orientation, plus the prior assumption of an upright head orientation (Mittelstaedt, 1983; Bringoux et al., 2003, 2004; Dyde et al., 2006; MacNeilage et al., 2007; De Vrijer et al., 2008; Zago, 2018).

Observers typically present a strong bias toward the direction of body rotation in estimating the orientation of a visual bar when their body is tilted >60° in the roll plane and in the absence of visual background information (the A-effect, Aubert, 1861). This deviation of SVV results from the under-compensation of body tilt (Van Beuzekom and Van Gisbergen, 2000). A static visual reference frame can reduce such bias in the perceived vertical (Haji-Khamneh and Harris, 2010). Moreover, also dynamic information about visual motion can reduce the bias contributing to SVV estimates. In one experiment, observers were presented with projectile motions of a visual target along parabolic trajectories with different orientations relative to physical gravity (Balestrucci et al., 2021). Participants were either upright or lying horizontally on their sides. When they were tilted, the bias in SVV was significantly reduced following the interception of parabolas aligned with the physical vertical.

Finally, vestibular stimulation resulting from increases of the gravito-inertial force (up to 1.4 *g*) with a short-radius centrifuge disrupts the time course of representational gravity, that is, the phenomenon in which the remembered vanishing location of a moving target is displaced downward in the direction of gravity, and more so with increasing retention intervals (De Sá Teixeira et al., 2017).

### A Neural Representation of “Visual” Gravity in The Vestibular Cortex

The evidence that somatosensory and vestibular signals can influence the visual perception of gravity-related information, raises the issue about the nature of the multimodal processing of sensory information taking place in the vestibular network. Until not long ago, the common view was that multisensory integration in the vestibular cortex would help resolve the ambiguities in the sensory signals. This could be instrumental to several higher-level processes afforded by vestibular information, such as spatial navigation, learning, and memory (Taube et al., 1996; Brandt et al., 2005; Taube, 2007; Gurvich et al., 2013; Smith and Zheng, 2013; Cullen and Taube, 2017), perceptual and motor decision making (Medendorp and Selen, 2017), mental imagery and mental rotation (Mast et al., 2006, 2014; Falconer and Mast, 2012), or bodily self-consciousness (Lopez, 2015, 2016).

**Figure 2:**
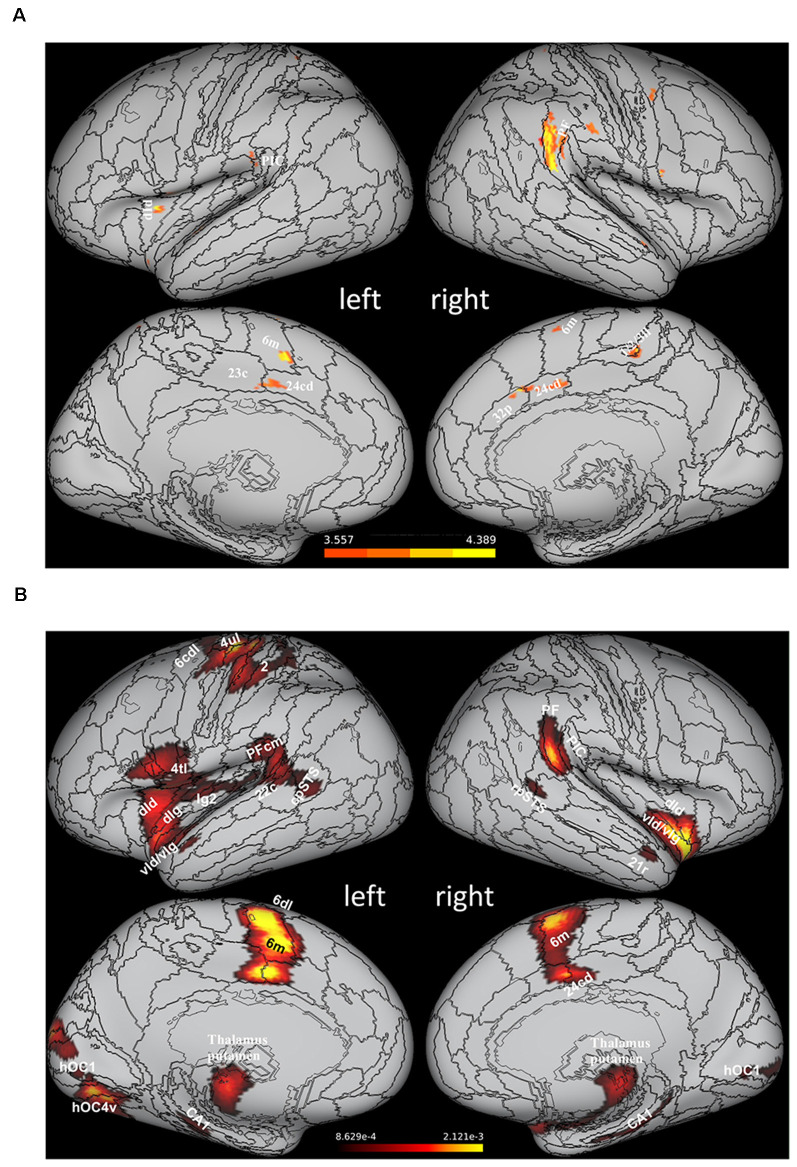
Areas of the vestibular network activated preferentially by stimuli congruent withthe effects of gravity. **(A)** Statistical activation mapresulting from the intersection of the brain activation map evoked by caloric stimulation and that derived by the statistical contrast between the activity evoked by visual motion congruent (1 *g*) and non-congruent (−1 *g*) with gravity (data from Indovina et al., 2005 replotted on the Conte69 inflated brain template). **(B)** Statistical activation map obtained with an ALE meta-analysis of 88 activation foci drawn from six studies reporting preferential fMRI activations in response to stimuli congruent with effects of gravity (Indovina et al., 2005, 2013b, 2016; Miller et al., 2008; Maffei et al., 2010; Rousseau et al., 2016). The activation map, overlapped onto the Conte69 inflated brain template (Glasser et al., 2016), was thresholded at voxel level (*p* < 0.05) and corrected at cluster level at *p* < 0.05 (Eickhoff et al., 2012). Given the limited number of foci and the compliant statistical thresholds used for the meta-analysis, this activation map should be considered for illustrative purposes rather than statistical ones in a strict sense. Labels correspond to: rostroventral area 40 (PIC), area supramarginalis (PF), area supramarginalis columnata magnocellularis (posterior; PFcm), ventral dysgranular and granular insula (vId/vIg), dorsal granular insula (dIg), dorsal dysgranular insula (dId), dorsal granular insula (dIg), granular insula 2 (Ig2), caudal dorsolateral area 6 (6 cdl), medial area 6 (6 m), area 4 (upper limb region, 4ul), area 4 (tongue and larynx region, 4tl), area 2 (2), caudal area 22 (22 c), caudal dorsal area 24 (24 cd), caudal area 23 (23 c), caudal dorsal area 24 (24 cd), posterior area 32 (32p), area1/2/3 (lower limb region, 1/2/3ll), rostral posterior superior temporal sulcus (rpSTS), caudal posterior superior temporal sulcus (cpSTS), cornu ammonis 1 (CA1), rostral area 21 (21r), human V1 (hOC1), human ventral V4 (hOC4v). PIC, posterior insular cortex.

Results of recent studies combining psychophysical and neuroimaging approaches have provided a complementary perspective on the function of the vestibular cortex by suggesting that multisensory processing in the vestibular network is directly concerned with gravity-related information. In an early study, Indovina et al. (2005) asked participants undergoing functional MRI scanning to perform a manual interception task with moving targets either congruent or not with natural gravity. Subjects’ interception timing was compatible with the use of *a priori* knowledge of gravity effects on the target motion (see above), and, most interestingly, fMRI data showed that visual targets congruent with natural gravity engaged preferentially cortical areas belonging to the vestibular network, as assessed by intersecting the statistical activation maps resulting from the contrast between the fMRI activations for natural and non-natural gravity with those obtained following vestibular caloric stimulation (Figure 2A). This result represented the first evidence that vestibular cortex activity can reflect processing of an internal representation of gravity effects on visual motion. Subsequent studies confirmed this result by integrating the visual paradigm used in Indovina et al. (2005) with manipulations of the visual background (Miller et al., 2008) and with apparent motion stimuli (Maffei et al., 2010). Two other studies by the same group extended the evidence to visual processing of self-motion, by showing that vestibular network areas could be activated, during visually simulated rollercoaster rides, by vertical motion congruent with gravity (Indovina et al., 2013b), as well as during a path integration task employing the same rollercoaster visual stimulation (Indovina et al., 2016). Finally, significant preferential activations of the posterior insular cortex have been reported for vertical compared to horizontal hand movements, particularly with the arm loaded so to enhance the effect of gravity on the hand motion (Rousseau et al., 2016). Figure 2B provides a graphical synopsis of these findings with a brain activation map obtained by performing an activation likelihood estimation (ALE) meta-analysis (Turkeltaub et al., 2002, 2012; Eickhoff et al., 2009) of 88 activation foci reported in these six studies (Indovina et al., 2005, 2013b, 2016; Miller et al., 2008; Maffei et al., 2010; Rousseau et al., 2016).

A particularly interesting aspect emerged from the aforementioned study by Miller et al. (2008), in that fMRI results indicated some of the vestibular network regions that may be specifically involved in extracting gravity cues from visual information. In these experiments, interception of vertical motion either congruent or not with the effects of gravity was performed with two visual scenarios, either a neutral background or a quasi-realistic scene incorporating static graphic elements, which provided reference and metric cues to scale the motion of the visual target to the overall scene size. It was found that the visual scene containing naturalistic pictorial cues facilitated the adoption of* a priori* knowledge of gravity to time the interception of the visual targets and that this process was associated with increased activity of the vestibular nuclei, of the nodulus and posterior cerebellar vermis. Thus, the extraction of gravity-related information from visual cues (which would help interpret the causality of the target motion to control predictively the timing of the interceptive action), might occur at rather early processing stages where vestibular and visual signals are first combined (see the introductory paragraph dealing with multisensory integration in the vestibular nuclei).

The extraction of gravity cues from visual signals can also be instrumental for the interpretation of biological motion. The neural correlates of this process have been investigated by an fMRI study, in which participants viewed intact or scrambled stick-figure animations of walking, running, hopping, and skipping recorded at either natural or reduced (Moon) gravity (Maffei et al., 2015). As was the case with inanimate object motion, the temporo-parietal junction (TPJ) and insular cortex were activated more strongly by viewing stick-figure animations recorded at natural compared to reduced gravity, supporting a role for these cortical regions in extracting gravity cues also from visual information related to biological motion. Cortical regions sensitive to biological motion configuration in the occipito-temporal cortex (OTC) showed a higher BOLD signal for reduced gravity compared to natural gravity, but with intact stick-figures only. Interestingly, connectivity analysis indicated significant modulation of the bi-directional connections between OTC and the peri-silvian regions involved in the internal representation of gravity, implying further that biological motion interpretation could depend on predictive coding of gravity effects (Maffei et al., 2015).

### Functional Parcellation of the Vestibular Network and Processing of “Visual” Gravity

The neuroimaging evidence discussed above underlines the complexity and heterogeneity of the brain areas comprising the vestibular network, hinting, in some cases, (see, for example, Miller et al., 2008) at potential differential functional properties with respect to the processing of gravity-related information. In fact, both anatomical and functional studies in the monkey brain indicate that the vestibular network may comprise at least two core regions, the parieto-insular vestibular cortex (PIVC), responding primarily to vestibular inputs, and the visual posterior sylvian area (VPS), which responds to both visual and vestibular inputs (Guldin and Grüsser, 1998; Chen et al., 2010). The putative human homologs of monkey PIVC and VPS have been identified, respectively, in the OP2 (Eickhoff et al., 2006)—a parietal operculum subregion responding mainly to vestibular and somatosensory stimuli, but also to visual motion in a small posterior subregion adjacent to the retroinsula (Mazzola et al., 2012; zu Eulenburg et al., 2012; Ibitoye et al., 2021)—and in a region of the supramarginal gyrus responding to vestibular and visual inputs, named posterior insular cortex (PIC; Sunaert et al., 1999; Beer et al., 2009; Frank et al., 2014; Frank and Greenlee, 2018). Although OP2 and PIC have been often considered a single functional region, generically labeled as human PIVC (Cardin and Smith, 2010; Riccelli et al., 2017a), recent evidence indicates that these two regions can be separated functionally at an individual subject level based on their fMRI responses to caloric vestibular stimulation and visual motion (Frank et al., 2016), implying that also the human vestibular network may comprise functionally distinct hubs.

**Figure 3:**
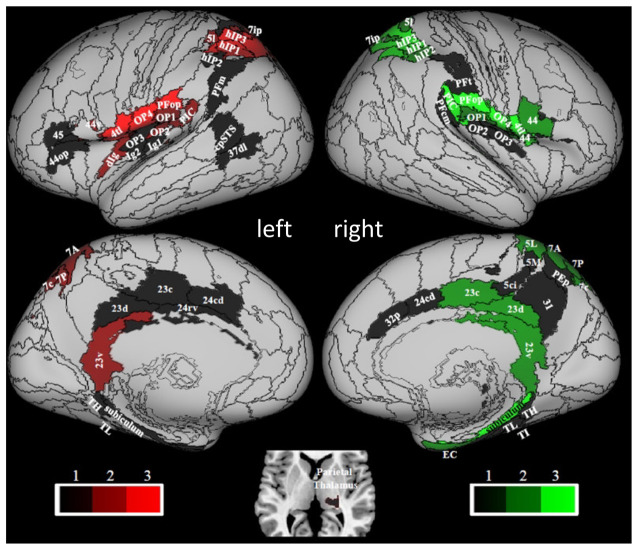
Hubness within the vestibular network computed by using data of 794 right-handed individuals drawn from the Human Connectome Project repository. PIC and OP2 are among the vestibular areas with the strongest hubness index. Red and green colors relate to regions in the left and right hemispheres, respectively. Brain regions are mapped onto the Conte69 inflated brain. Labels correspond to: parietal opercula 1, 2, 3, 4 (OP1, 2, 3, 4), caudal posterior superior temporal sulcus (cpSTS), area 4 tongue and larynx region (4 tl), granular insula 2 (Ig2), granular insular 1 (Ig1), Area supramarginalis opercularis (PFop), rostroventral area 40 (PIC), area supramarginalis columnata magnocellularis (posterior; PFcm), area supramarginalis tenuicorticalis (PFt), area supramarginalis magnocellularis (PFm), caudal posterior superior temporal sulcus (cpSTS), dorsolateral area 37 (37 dl), human intraparietal 1, 2, 3 (hIP1, 2, 3), lateral area 5 (5I), intraparietal area 7 (7ip), superior parietal lobe (5 ci), area 31 (31), dorsal area 23 (23 d), caudal area 23 (23 c), ventral area 23 (23v), caudodorsal 24 (24 cd), pregenual area 32 (32p), ventral area 44 (44v), rostral area 45 (45), opercular area (44op), dorsal granular insula (dIg), granular insula 2 (Ig2), hippocampotemporalis (TH), subiculum, latero posterior parahippocampal gyrus (TL), temporal agranular insular cortex (TI), entorhinal cortex (EC). Figure drawn from Indovina et al. (2020).

The results of a structural connectivity study by Indovina et al. (2020) are compatible with this view. By drawing data from 974 subjects of the repository of the Human Connectome Project, it was found that the structural connectivity pattern of PIC was consistent with a prominent role in visuo-vestibular processing, whereas that of OP2 was consistent with the integration of mainly vestibular, somato-sensory, and motor information (Figure 3). From the analyses reported in that article, in fact, PIC showed bilateral connections with the medial superior parietal regions including VIP (7r, 7ip) and with most of the thalamus, and ipsilateral connections with the insula, peri-sylvian regions, frontal premotor regions, occipital and temporal areas, the posterior cingulate cortex, and the rostral hippocampus. Conversely, OP2 showed ipsilateral connections with the rest of the insula and the peri-sylvian region, the superior parietal cortex including VIP (A7r, A7ip), and the somatosensory cortex. Furthermore, the brain areas connected with PIC were more diffuse and bilateral compared to the brain areas connected with OP2. Remarkably, these structural connectivity patterns are in line with those reported by neuroanatomical tracing studies in the squirrel monkeys for VPS (area T3) and PIVC, respectively. Indeed, in these monkeys, VPS shows strong connections with parieto-occipital and parieto-temporal regions (area 19), the upper bank of the temporal sulcus (STS-area), anterior cingulate gyrus, and parts of the posterior parietal area 7, while PIVC is connected primarily with Brodmann’s areas 8a, 6, 3a, 3aV, 2, and posterior parietal area 7ant (Guldin et al., 1992).

Aside from the identification of these two main hub regions, another aspect of the organizational scheme of the vestibular network considered by Indovina et al. (2020), was the possible lateralization of vestibular functions, as PIC and OP2 structural connectivity patterns were found to be lateralized to the left hemisphere, whereas those of the posterior peri-sylvian supramarginal and superior temporal gyri were lateralized to the right hemisphere. Moreover, these lateralization effects did not depend on handedness. Evidence in the literature with respect to the lateralization of the fMRI responses observed in vestibular areas following vestibular stimulation and of their functional connectivity, however, appears far from conclusive. Early studies indicated that vestibular fMRI activations following vestibular stimulation may be lateralized to the right hemisphere in right-handed individuals and to the left hemisphere in left-handed people (Dieterich et al., 2003; Janzen et al., 2008; Lopez et al., 2012; Kirsch et al., 2018). Along these lines, an ALE meta-analysis of fMRI activations evoked by caloric, galvanic, and sound-evoked vestibular stimulation showed larger activation volumes in the parietal, temporal, and insular cortices of the right hemisphere during stimulation of the right ear than in the left hemisphere following stimulation of the left ear (Lopez et al., 2012). However, because of the low spatial resolution of the ALE meta-analysis technique, laterality differences in the posterior peri-sylvian cortex could not be assessed. Furthermore, a recent study on OP2 connectivity and fMRI activation following caloric stimulation in healthy subjects and patients affected by vestibular neuritis failed to show any lateralization in OP2 functional connectivity or in its response to caloric stimuli. Nevertheless, it pointed out that the effects of the peripheral vestibular disease were asymmetrical and the relationship between activity and dizziness/visual dependence was observed only in the right hemisphere, suggesting right lateralization of higher-order vestibular functions (Ibitoye et al., 2021). Conversely, a systematic review of the clinical outcomes of insular infarction concluded that despite vestibular-like syndromes being reported more often after right insular stroke, a clear lateralization has not yet clearly emerged for the Vestibular-like Syndrome (Di Stefano et al., 2021). Overall, this fragmented evidence in the literature for lateralization patterns may indicate another level of anatomo-functional compartmentalization within the vestibular network, but further studies are still needed to draw definite conclusions on the degree and type of lateralization of vestibular functions.

**Figure 4:**
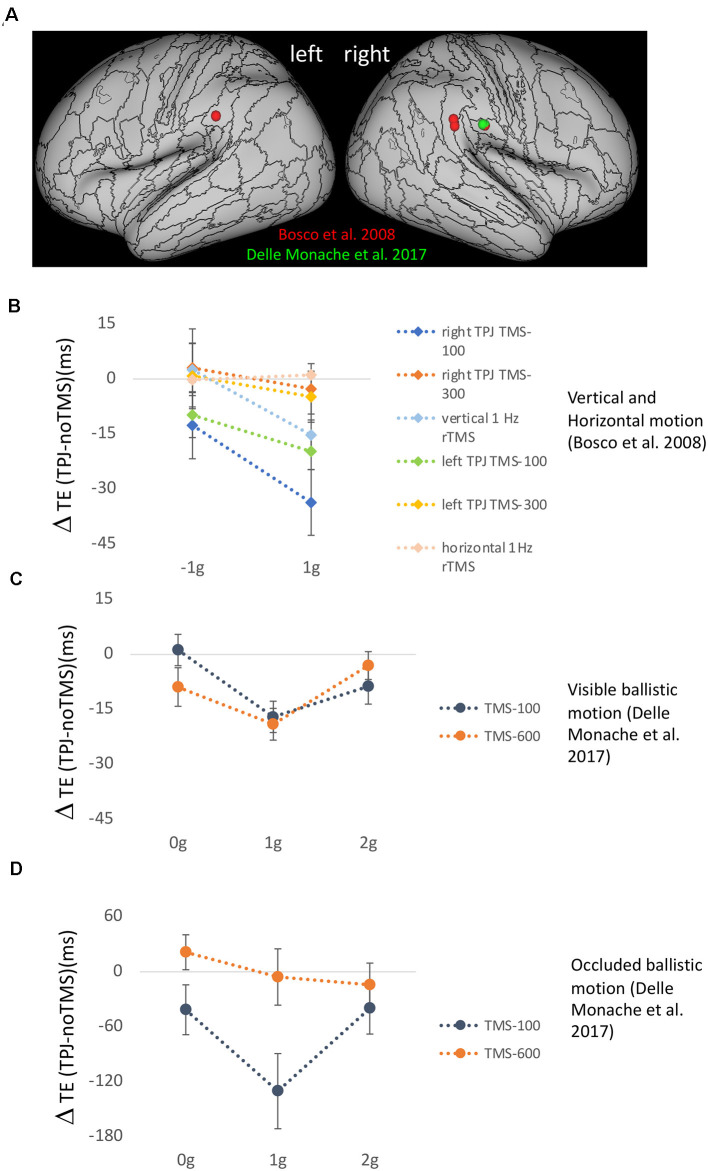
**(A)** Effects of transcranial magnetic stimulation (TMS) of temporo-parietal junction (TPJ) on the timing of manual interception responses. **(A)** Anatomical location of the mean stimulation sites onTPJ reported for the experimental protocols of Bosco et al. (2008) (red) and Delle Monache et al. (2017) (green). **(B)** Mean timing error differences (±SEM) observed following TPJ stimulation compared to trials without TMS, for 1 *g* accelerated and decelerated motion in the six experimental protocols of Bosco et al. (2008). All conditions involved vertical target motion except that labeled horizontal 1 Hz rTMS. **(C)** Mean timing error differences (±SEM) observed following TPJ stimulation compared to trials without TMS for unperturbed 1 *g* and perturbed 0 *g* and 2 *g* ballistic trajectories, which were visible throughout their extent (Experiment 2 of Delle Monache et al., 2017). **(D)** Mean timing error differences (±SEM) observed following TPJ stimulation compared to trials without TMS, for unperturbed 1 *g* and perturbed 0 *g* and 2 *g* ballistic trajectories, which were occluded 500 ms after the perturbation until landing (Experiment 1 of Delle Monache et al., 2017).

The functional parcellation within the vestibular network in relation to the strong hubness shown by PIC and OP2, instead, may suggest a potential role in the processing of gravity-related visual information for the component of the vestibular network integrating mainly visual and vestibular information, that is, the nodal area PIC and its interconnected areas. The results of three studies involving transcranial magnetic stimulation (TMS) of cortical sites in TPJ of the visual-vestibular network provide further support to this idea (Bosco et al., 2008; Delle Monache et al., 2017; De Sá Teixeira et al., 2019). In the first two studies, TPJ activity, as well as that of visual motion area hMT/V5+ and of the intraparietal sulcus (IPS), involved in visuomotor control, was disrupted by means of various online and off-line TMS paradigms, while healthy participants intercepted target motion either congruent or not with the effects of natural gravity (Figure 4). In the first study, targets moved vertically downward either accelerated by gravity or decelerated by the same amount (Bosco et al., 2008). In the second study, participants intercepted computer-simulated baseball fly-ball trajectories, which could be perturbed or not with the effects of altered gravity (either constant velocity, 0 *g*, or accelerated 2 *g*) and occluded 500 ms after the perturbation until landing (Delle Monache et al., 2017). A common finding across studies was that TPJ stimulation affected selectively the timing of the interceptive responses to visual motion congruent with the effects of gravity. Conversely, TMS applied on area hMT/V5+ altered the interceptive responses to all types of motion (not shown in Figure 4). Interestingly, the effects of stimulation of both cortical sites on the timing of the interceptive responses were restricted to specific temporal windows during the target motion trajectory. In Bosco et al. (2008), two TMS pulses (dpTMS) were delivered either 100 or 300 ms after the onset of the vertical trajectories (trajectory durations comprised between 700 and 890 ms), and significant effects of TPJ and hMT/V5+ stimulation were evident only for the earliest time window, implying that processing of visual information about the very beginning of the target trajectory in these two cortical areas is causally related to the timing of the interceptive action (see Figure 4B). With respect to the specific contribution of TPJ to the interceptive timing, the selectivity of the effects of its disruption for target motion congruent with natural gravity goes along with the idea that this cortical region is responsible for processing gravity-related visual information and contributes to an internal representation of gravity effects. This interpretation is supported also by the results of the second study with ballistic trajectories. In these experiments, three TMS pulses (tpTMS) were delivered 100 ms after either the perturbation or the occlusion of the visual motion on TPJ, hMT/V5+, and IPS sites. Once again, TPJ stimulation affected selectively the timing of the interceptive responses to unperturbed fly-ball trajectories, which were congruent with the effects of gravity and air friction, whereas stimulation of visual motion area hMT/V5+ altered the interceptive timing regardless of the type of motion trajectory. Remarkably, statistically significant stimulation effects for these two cortical regions were evident only when tpTMS was delivered at the onset of the trajectory perturbation (or at corresponding time frames in the unperturbed trajectories), with the target visible, but not when tpTMS was delivered just after the target disappearance (see Figures 4C,D). This result, while strengthening the idea that TPJ activity is causally related to the processing of gravity information embedded in visual signals, makes it unlikely that it may be also engaged in motion extrapolation. Instead, consistent with previous electrophysiological and neuroimaging evidence indicating a putative role of IPS in motion extrapolation (Assad and Maunsell, 1995; Lencer et al., 2004; Olson et al., 2004; Ogawa and Inui, 2007; Shuwairi et al., 2007; O’Reilly et al., 2008; Beudel et al., 2009; Makin et al., 2009), IPS stimulation was effective at both temporal windows, however altering the timing of the interceptive responses only for trajectories incongruent with natural gravity (Delle Monache et al., 2017). Therefore, it remains unclear which neural structures, likely belonging to the vestibular network, may participate in the extrapolation of natural gravitational motion, at least within the behavioral context examined by these studies.

The third TMS study, carried out by De Sá Teixeira et al. (2019), extended to the perceptual domain the evidence regarding the putative role of TPJ in the processing of the internal representation of gravity. The experiments were aimed at elucidating the potential neural basis for the observed phenomena of representational momentum and representational gravity, that is, the forward and the downward perceived vanishing location of a moving target (Freyd and Finke, 1985; Hubbard, 1990). To this end, offline continuous theta-burst stimulation (cTBS) was used to depress the excitability of TPJ and visual motion area hMT/V5 before the execution of a standard spatial localization task. The study reported an increase in representational gravity following disruption of hMT/V5+ activity and an increase of representational momentum following TPJ stimulation. These results are compatible with a push-pull mechanism between the relative contributions of area hMT/V5+ and TPJ. Accordingly, the spatial localization responses might be determined by the reciprocal balance between perceived kinematics and anticipated dynamics (i.e., the effects of gravity acceleration).

Overall, these three TMS studies have established a causal relationship between the activity of TPJ and the use of *a priori* knowledge of gravity engaged by visual motion information.

### What Can Be Learned From Patients’ Studies?

Further insight on the role of the vestibular cortex in the processing of gravity-related information has come from studies involving stroke patients with lesions of the peri-sylvian areas belonging to the vestibular network. Blood supply to the vestibular network largely depends on branches of the middle cerebral artery (MCA), a vessel frequently involved in acute stroke (cf. Ng et al., 2007). Common vestibular symptoms like vertigo, dizziness, and postural instability have often been reported following MCA infarction, particularly if lesions include the putative human homolog of PIVC (Marsden et al., 2005; Eguchi et al., 2019; Di Stefano et al., 2021). In addition, strokes involving the insula and TPJ have been linked to deficits of awareness, in line with the role played by these regions in providing an anchor for self-location and first-person perspective (Pfeiffer et al., 2014; Ferrè and Haggard, 2016; Lopez, 2016).

Functionally, many symptoms associated with damages to the vestibular network can be interpreted as failures to process or integrate information derived from multiple sensory sources and/or to reconcile these inputs with prior information resulting from a lifelong experience with gravity. In consequence of these unsolved conflicts, brain-damaged patients can experience pathological tilts in perceived verticality (Karnath, 2007; Baier et al., 2012; Pérennou et al., 2014; Dieterich and Brandt, 2019) or more complex sensations such as paroxysmal tilts of the visual scene, (e.g., room tilt illusion, Tiliket et al., 1996; Malis and Guyot, 2003; Sierra-Hidalgo et al., 2012) and altered sense of embodiment (Blanke et al., 2004; Bünning and Blanke, 2005; Lopez et al., 2008). Paradigmatic conditions are cases when patients refer to transitory and often dramatic perturbations of the perceived upright posture, which is felt as no longer aligned with the gravitational vector and/or the subjective sense of self. In contraversive pushing, for example, patients spontaneously sit or stand with their longitudinal body axis tilted toward the paretic side, and actively use the non-paretic limbs to push away from the non-paretic side (cf. Davies, 1985; Karnath, 2007; Pérennou et al., 2008; Baier et al., 2012). This unusual behavior mainly emerges in cases of damage to regions involved in processing body perception and graviceptive information, such as the posterior thalamus and parts of the insula, the superior temporal gyrus, and post-central gyrus (Karnath, 2007; Pérennou et al., 2008; Baier et al., 2012). The capacity to determine the vertical orientation of the visual surrounding is often spared, suggesting that pushing could reflect the patient’s attempts to compensate for a mismatch between the perceived postural and visual vertical (Karnath et al., 2000) or to align the body with the verticality reference (Pérennou et al., 2008). On the other hand, lesions extending to TPJ have been associated with feelings of disembodiment, i.e., the paradoxical, temporary sensation of being localized elsewhere with respect to one’s physical body (Blanke et al., 2004; Bünning and Blanke, 2005). These out-of-body experiences (OBEs) are often accompanied by vestibular sensations such as feelings of flying or floating (Blanke et al., 2004), and are likely linked to two disturbances: a failure to integrate inputs relative to the body from different sensory channels, and vestibular dysfunction. The former would cause what has been described as “disintegration” in personal space and could explain the illusory reduplication of the experient’s body. The latter would further affect the integration between the central representations of the body and extra-personal space (possibly at the TPJ), producing the experience of seeing oneself from an elevated position (Blanke et al., 2004).

**Figure 5:**
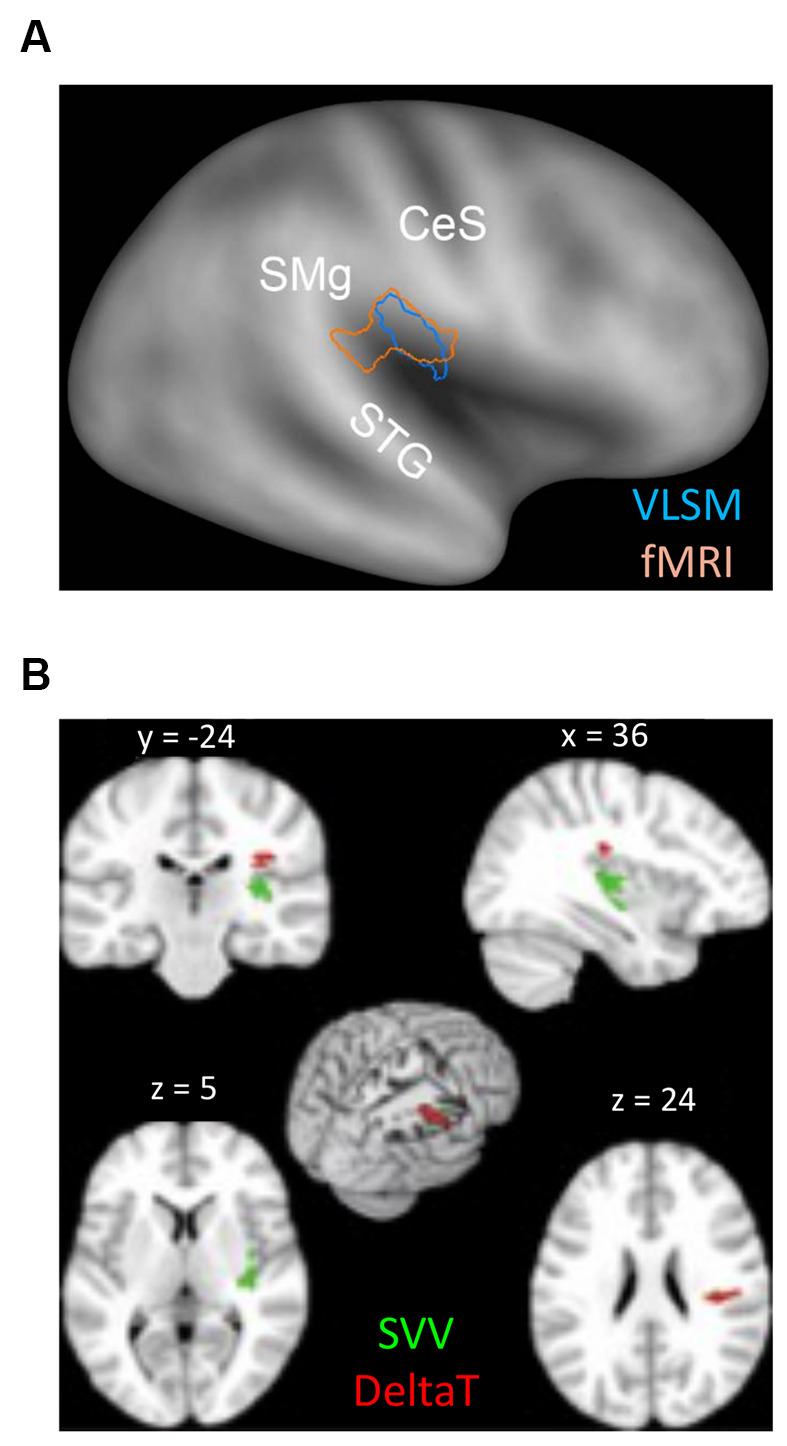
**(A)** Overlap between the peri-sylvian region activated by the contrast (1 *g* > −1 *g*) in the fMRI study on healthy subjects (pink contour) and the lesion map resulting from the VLSM analysis performed by Maffei et al. (2016) (see text), which considered the lesioned brain regions associated with higher DeltaT values (blue contour). Contours are plotted onto the PALS inflated human brain template (Caret). CeS, Central Sulcus; STG, Superior Temporal Gyrus; SM, Supramarginal Gyrus. **(B)** Lesion Subtraction Map reported by Maffei et al. (2016). Red voxels were found to be damaged more frequently (>65%) in patients with the highest values of DeltaT (*n* = 7) than in the seven patients with the smallest DeltaT values. Green voxels were found more frequently damaged (>65%) in patients with altered SVV estimation than in the five patients showing the smallest DeltaT values and intact SVV estimation. The MNI coordinates are reported on top of each brain section. Modified from Maffei et al. (2016). Cortex with permission of Roopa Lingayath, Senior Copyrights Coordinator ELSEVIER. SVV, subjective visual vertical.

In contrast to the many descriptions of altered perception of verticality and/or body orientation, less is known about how lesions to the vestibular network affect interactions with moving objects. As reviewed above, successful planning of interception movements takes advantage of an internal model of gravity effects stored in the vestibular cortex, which is used to supplement the continuous flux of information conveyed by the sensory channels (McIntyre et al., 2001; Indovina et al., 2005). To explore this issue, one study investigated the capacity to efficiently intercept a moving target in patients diagnosed with MCA infarction (Maffei et al., 2016), by employing a similar task to the one used in Indovina et al. (2005). Maffei and collaborators considered the DeltaT, i.e., the relative difference between timing errors of the responses to the two types of target motion (1 *g*, −1 *g*), as an indicator of whether patients’ interceptive responses reflected or not *a priori* assumptions of gravity effects. In fact, if 1 *g* and −1 *g* trials were to be correctly discriminated, DeltaT would be expected to be small, timing errors being similarly small in both conditions. Conversely, if priors about gravitational acceleration are being applied to both types of motion, responses to −1 *g* trials should be anticipated (Zago et al., 2004) producing an increase in DeltaT. Consistent with these assumptions, an abnormally large DeltaT was found in a subgroup of patients. Correlation with neuroanatomical data *via* voxel-based lesion-symptom mapping VLSM (Bates et al., 2003) and lesion subtraction analyses showed an association with damage to peri-sylvian areas, centered in the parietal operculum. In healthy subjects, this same region has been found activated in fMRI studies comparing 1 *g* and −1 *g* motion (Maffei et al., 2010), suggesting a role of this region in discriminating between motions that either obey or violate gravity (Figure 5A). On this basis, it has been postulated that, by losing this ability, stroke patients could not detect the mismatch between incoming sensory signals and expectations based on stored models of gravity, thus failing to apply the correct model to each type of motion. Remarkably, this study reported also that patients with large DeltaT showed a relatively intact verticality perception. Compared to interception, estimation of the subjective visual vertical (SVV) requires aligning the perceived vertical estimation with the veridical vertical, i.e., it involves spatial rather than temporal processing of gravity information, two operations that may not rely on the same neural substrates (Figure 5B). In fact, SVV impairments are more frequently reported following strokes to the posterior insula (Brandt et al., 1994; Baier et al., 2013; Maffei et al., 2016) as are the postural disturbances associated (Dieterich and Brandt, 2019), suggesting again possible dissociations as to where and how gravity information is processed. Indeed, in the afore-mentioned study by Maffei et al. (2016), VLSM analysis indicated that greater deviations of SVV were associated preferentially with lesions mainly centered on the posterior insula, that is, in a site distinct from the parietal operculum, which was preferentially associated with impairment of discrimination of gravitational motion.

Another clinical disorder providing insights on the study of the internal representation of gravity in the vestibular cortex is functional dizziness, that is, chronic dizziness without an organic cause. This disorder has recently been defined as persistent postural-perceptual dizziness or PPPD (Staab et al., 2017). One of the possible causes of PPPD might be the behavioral maladaptive shift to visual dependence, with greater reliance on visual rather than vestibular information for spatial orientation, which persists even after the resolution of the acute vestibular problem (Cousins et al., 2014). One possibility entertained by a recent fMRI study (Riccelli et al., 2017b) is that this greater reliance on visual information by PPPD patients might be paralleled by a lower reliance on* a priori* information about gravity stored in the vestibular cortex. Thus, fMRI signals were acquired during visually simulated rollercoaster rides along vertical and horizontal directions in 14 patients with PPPD secondary to an acute peripheral vestibular episode, like vestibular neuritis (VN) or benign paroxysmal positional vertigo (BPPV), as well as in healthy controls (Riccelli et al., 2017b). PPPD patients who had suffered from vestibular neuritis underwent caloric testing in the acute stage of the peripheral vestibular disease and 6 months later to evaluate the extent of their recovery. Patients who experienced benign paroxysmal positional vertigo as a trigger for PPPD had no symptoms or signs of active positional vertigo at the time of entry in the study. Statistical comparisons between the fMRI activation maps observed during the vertical vs. the horizontal self-motion bouts showed a significant decrease of the BOLD signal in the right middle insula in the group of PPPD patients as compared with the healthy controls. In the light of the consistent reports that the insular cortex is activated preferentially by visual motion congruent with the effects of gravity (see above, and also Figure 2), this result appears to be in line with the idea that PPPD patients rely to a lesser extent than healthy subjects on internalized gravity-related information. However, it is also worth noting that this result has been obtained by collapsing data for the direction of motion (vertical vs. horizontal) regardless of the kinematics (accelerated/decelerated at 1 *g*, constant speed), Thus, more controlled studies are needed to disentangle whether this decrease in the activity of the right insula shown by PPPD patients is related to the internal representation of gravity, for example by combining neuroimaging and psychophysical approaches in these patients in order to measure both interception and fMRI responses to visual motion either coherent or incoherent with gravity effects.

### Concluding Remarks

We reviewed experimental evidence gathered from behavioral, neuroimaging, and patients’ studies in support of the hypothesis that an internal model estimating the effects of gravity on visual objects is constructed by transforming vestibular estimates of physical gravity, processed in the brainstem and cerebellum, into an internalized supramodal representation of gravity stored in the vestibular network. The integration of the internal model of gravity with visual and other non-vestibular signals can take place at multiple levels in the areas of the vestibular network and might be instrumental in extracting gravity cues from sensory signals, such as retinal ones, that may not relate directly to physical gravity. This process would afford the implicit interpretation of a virtual reproduction of the physical world, like that rendered by a movie. Although it seems reasonable to consider this process as distributed among the brain areas belonging to the vestibular network, we suggest that brain regions more closely associated with PIC could provide a stronger contribution, by virtue of their denser reciprocal connectivity with cortical areas engaged in the processing of spatio-temporal features of the visual stimuli (Indovina et al., 2020).

## Author Contributions

SD, II, MZ, ED, FL, and GB drafted the manuscript. All authors contributed to the article and approved the submitted version.

## Conflict of Interest

The authors declare that the research was conducted in the absence of any commercial or financial relationships that could be construed as a potential conflict of interest.

## Publisher’s Note

All claims expressed in this article are solely those of the authors and do not necessarily represent those of their affiliated organizations, or those of the publisher, the editors and the reviewers. Any product that may be evaluated in this article, or claim that may be made by its manufacturer, is not guaranteed or endorsed by the publisher.
